# Determination of Gingival Index and Salivary Proinflammatory Cytokines (Interleukin 6 and Tumour Necrosis Factor α) After Chemical–Mechanical Retraction Procedure

**DOI:** 10.3390/diagnostics15040424

**Published:** 2025-02-10

**Authors:** Marko A. Igić, Milena M. Kostić, Nadica S. Đorđević, Jelena T. Bašić, Marija G. Đorđević, Nikola R. Gligorijević, Goran Jovanović, Rodoljub G. Jovanović, Natasha Stavreva, Jelena T. Todić, Jana Pešić Stanković, Simona M. Tarana Stojanović

**Affiliations:** 1 Clinic for Dental Medicine, Faculty of Medicine, University of Niš, 18000 Niš, Serbia; marko.igic@medfak.ni.ac.rs (M.A.I.); milena.kostic@medfak.ni.ac.rs (M.M.K.); marija.jovanovic@medfak.ni.ac.rs (M.G.Đ.); nikola.gligorijevic@medfak.ni.ac.rs (N.R.G.); goran.jovanovic@medfak.ni.ac.rs (G.J.); simona.stojanovic@medfak.ni.ac.rs (S.M.T.S.); 2 Department of Dentistry, Faculty of Medicine, University of Priština in Kosovska Mitrovica, 38220 Kosovska Mitrovica, Serbia; jelena.todic@med.pr.ac.rs; 3 Department for Biochemistry, Faculty of Medicine, University of Niš, 18000 Niš, Serbia; jelena.basic@medfak.ni.ac.rs; 4 Faculty of Medicine, University of Niš, 18000 Niš, Serbia; rodoljubj97@gmail.com; 5 Faculty of Dentistry, University Cyrill and Methodius, 1000 Skopje, North Macedonia; nstavreva@stomfak.ukim.edu.mk; 6 Public Health Institute Niš, Faculty of Medicine, University of Niš, 18000 Niš, Serbia; jana.pesic.stankovic@medfak.ni.ac.rs

**Keywords:** retraction procedure, gingival index, proinflammatory cytokines

## Abstract

**Background and Objectives:** The aim of this study was to test the impact of the gingival chemical–mechanical retraction procedure on the value of the gingival index (GI) and salivary concentrations of interleukin 6 (IL-6) and tumour necrosis factor α (TNF-α). The importance of this clinical study is that it provides an exact conclusion regarding the behaviour of the gingival tissue after the application of an impregnated retraction cord in the gingival sulcus of the subjects. **Materials and Methods:** The research was conducted as a prospective clinical study. The study included 60 subjects divided into two experimental groups, i.e., with and without tooth preparation. Each group was divided into two subgroups according to the retraction agent used. The parameters were measured before, one day after, and three days after the retraction procedure. **Results:** One day after the application of both gingival retraction agents in both study groups, there was a statistically significant increase in the value of the GI and the salivary concentrations of IL-6 and TNF-α (*p* < 0.001) compared to the values at the beginning of the study. After 72 h, the values decreased in comparison to those from the second observation period, but they remained statistically significantly higher than the initial values of the study (*p* < 0.001). **Conclusions:** The study showed moderate inflammation of the gingival tissue one day after the retraction procedure, which decreased over time. Higher values of the studied parameters were observed with the application of the ferric sulphate-based retraction agent.

## 1. Introduction

The effect of the interaction of mechanical pressure and chemical activity on the gingiva tissue using the chemical–mechanical gingival retraction method leads to the desired widening of the gingival sulcus and a decrease in fluid secretion in it; therefore, the demarcation line of preparation can be accurately imprinted. This guarantees a subsequent tight placement of the artificial crown on the tooth tissue, with maximum respect for the integrity of the periodontal tissue [1,2].

The most commonly used gingival retraction agents are metal salts (aluminium, iron, zinc) with astringent action [3,4]. The literature data have shown that astringents, which act by protein precipitation and inhibition of the transcapillary movement of plasma proteins, possess a certain aggressiveness that may cause tissue damage during the retraction procedure [5,6].

To date, the majority of studies dealing with the adverse effects of astringent retraction solutions were performed under in vitro conditions [7,8,9]. The results of these studies indicate the existence of cytotoxic and inflammatory effects of these compounds. This highlights the importance of a clinical study that, by monitoring the clinical objective parameters of gingival damage (gingival indices), as well as by analysing the concentration of inflammation markers (proinflammatory cytokines), provided an exact conclusion regarding the behaviour of the gingival tissue after the application of an impregnated retraction cord in the gingival sulcus of the subjects.

As a consequence of the described clinical process, gingivitis appears as a reversible inflammation that occurs due to bacterial colonization and the formation of an oral biofilm. Bacterial products lead to the activation of immune system cells, i.e., monocytes/macrophages, which further secrete inflammatory mediators and the cytokines interleukins 1 and 6 (IL1, IL-6), tumour necrosis factor-alpha TNFα, and the release of matrix metalloproteinase enzymes (MMPs) [10].

Proinflammatory cytokines, such as TNF-α and IL-6, can be defined as molecules essential in triggering and maintaining inflammatory and immunological responses [11,12]. From the viewpoint of oral medicine, proinflammatory cytokines are associated with periodontal tissue destruction, proteinase induction, and bone decomposition; thus, an increase in their levels in gingival fluid and saliva is an important diagnostic indicator [13,14].

The aim of this study was to test the impact of the chemical–mechanical retraction procedure on the value of the GI and salivary concentrations of IL-6 and TNF-α. This research is based on the assumption that the chemical–mechanical retraction procedure leads to increased values of the studied parameters.

## 2. Materials and Methods

The study was conducted at the Clinic for Dental Medicine in Niš. All subjects were informed of the purpose of the study and signed written consent forms. The study was conducted in accordance with the provisions of the Declaration of Helsinki and approved by the Ethics Committee of the Faculty of Medicine, University of Niš (12-1550/9).

### 2.1. Subjects and Examined Agents

The research was conducted in a clinical prospective study. It was conducted on 60 subjects divided into four groups. The group of subjects was homogeneous, and the study included healthy subjects, non-smokers, aged 20–40 years, without pathological changes in the oral cavity, with a healthy periodontium, and without carious lesions. Before the start of the intervention, all subjects underwent a clinical examination and assessment of the state of their gingival health (gingival index, gingival bleeding index), i.e., the health of the periodontium (CPITN) [15].

The sample size was calculated using the commercial statistical program G*Power for two-way null hypothesis testing. The F-test and ANOVA (analysis of variance) were used to determine the sample size in this program package. The following parameters were set: probability of type 1 error α = 0.05 and a study power of 0.8. With such initial parameters and based on the publication of Di Venera et al., the smallest sample size of 8 subjects per group was obtained (Institute of Public Health, Niš, Serbia).

The subjects were divided into two experimental groups (G1—respondents for whom the making of one artificial crown was indicated (n = 30) and G2—respondents for whom prosthetic therapy was not indicated (n = 30)). Each group was further divided into two subgroups according to the type of gingival retraction agent most commonly used in clinical practice, i.e., R1: 25% AlCl_3_ (aluminium chloride)—Racestyptin Solution (Septodont, Saint Maur des Fosses, France) and R2: 15.5% Fe_2_(SO_4_)_3_ (ferric sulphate)—Astringedent (Ultradent Products Inc., South Jordan, UT 84095-3942, USA).

### 2.2. Gingival Index—GI

Numeric values of GI ranging from 1 to 4 determine the clinical condition of the gingiva, depending of the severity of inflammatory changes. The GI was obtained by summing the index values from all axial sides of the teeth and then dividing the obtained value by 4 [16].

### 2.3. Biochemical Analysis of the Levels of Proinflammatory Cytokines

Salivary IL-6 levels were determined using the sandwich enzyme-linked immunosorbent assay (ELISA; R&D Systems, Abingdon, UK). The minimum detectable dose (MDD) was 0.7 pg/mL. Salivary TNF-α levels were determined using the sandwich enzyme-linked immunosorbent assay (ELISA; R&D Systems, Abingdon, UK). The minimum detectable dose (MDD) was 6.23 pg/mL. The tests were performed following the manufacturer’s instructions [17].

### 2.4. Clinical Procedure

The study period included three observation periods: prior to (T0), 24 h after (T1), and 72 h (T2) after the chemical retraction procedure.

The observation period T0 revealed the condition of the gingiva before treatment, and the periods T1 and T2 were the periods in which pathological changes were expected, based on the literature data [7,18].

At the beginning of the study (T0), the GI in group G1 was determined for the tooth indicated for preparation, i.e., artificial crown-making. In G2 subjects, the index was determined on the upper left central incisor. A sterile tube was used for sampling non-stimulated saliva from the subjects of both experimental groups [18].

After sample collection, the subjects from group G1 underwent tooth preparation, with maximum preservation of gingival integrity. The preparation demarcation was located 0.25–0.5 mm in the gingival sulcus [15].

The chemical–mechanical gingival retraction procedure included the application of the retraction cord (Elite Cord, Zhermack SpA, Badia Polesine (RO), Italy) of 00 diameter, soaked in one of the two studied gingival retraction agents (R1 and R2), in both groups of subjects for 5 min, without using force [19]. In group G1, the cord was applied into the gingival sulcus of the prepared tooth, and in in group G2, into the gingival sulcus of the upper left central incisor, using a plastic filling instrument. All subjects were observed by the same examiner.

In other observation periods (T1 and T2), the GI for the reference teeth was determined in both observation groups. Non-stimulated saliva samples were obtained in sterile tubes. All saliva samples were centrifuged at 10,000 rpm, for 5 min (Allegra 64R centrifuge, Beckman Coulter, Inc., Brea, CA 92821, USA). The separated supernatant was frozen at −80 °C until the analysis [1,20].

The creation of artificial crowns for the prepared teeth occurred following the end of the study period.

### 2.5. Statistical Analysis

The acquired data was statistically analysed using SPSS 15.0 software (SPSS Inc., Chicago, IL, USA). The Mann–Whitney U test was used to determine the significance of the difference (*p*) between the continuous variables in the two separate subject groups. The significance of the differences of the continuous variables within groups was analysed using the Student’s *t*-test for paired samples or the Wilcoxon signed ranks test, depending on the normality of data distribution. The Shapiro–Wilk test was performed as a test of normality. Statistical significance was defined as a value of *p* < 0.05.

Analyses of variance for repeated measures (RM ANOVA) were used to examine changes in the arithmetic mean of the variables measured during the three observation periods in the study groups. Using the partial eta squared (p2) values, the effects of the adjustments were classified as modest for parameter values greater than 0.01; medium for values greater than 0.06; and large for values greater than 0.14. Statistical significance was defined as a value of *p* < 0.05.

## 3. Results

A total of 24 h after the application of both gingival retraction agents in both study groups, there was a statistically significant increase in the GI values and salivary concentrations of IL-6 and TNF-α (*p* < 0.001) compared to the values at the beginning of the study. The values at 72 h were statistically significantly greater than those at the start of the study (*p* < 0.001), although they were lower than those at the second observation period (Table 1, Table 2 and Table 3).

The effects within the groups were monitored to determine whether there was a statistically significant change in the GI for the given group of subjects (R1 + R2), as well as for the subgroups in which a particular retraction agent was used (R1 or R2) (Table 4).

Evaluating the impacts among the examined subgroups (between participant effects) revealed a statistically significant difference in GI values over the study duration (*p* < 0.001).

Alterations in GI values throughout the study period showed a statistically significant difference exclusively in G2, based on the type of retraction agent used.

In groups G1 and G2, a significant statistical increase in IL-6 concentration (*p* < 0.001) was observed throughout the study period, with a substantial effect incurred according to the retraction agent employed. A somewhat reduced level of statistical significance for the rise in the concentration of IL-6 was noted in the subgroup of participants with unprepared teeth (G2), for which a ferric sulphate-based retraction agent was employed (R2) (*p* < 0.01) (Table 5).

Testing the effect of the retraction agent type on the salivary concentration of IL-6 did not reveal any statistically significantly differences between the subgroups over the entire study period. In general, a more significant effect of the retraction agent on the concentration of IL-6 was observed in G1.

Changes in the concentration of IL-6 during the study period in the subgroups of the prepared teeth group (G1) occurred in a statistically significantly different manner, depending on the type (*p* < 0.01), unlike the results for the G2 group.

A statistically significant increase in the salivary concentration of TNF-α during the study period was recorded in groups G1 and G2 (*p* < 0.001), except in the group with unprepared teeth (G2), in with ferric sulphate applied (R2), for which *p* < 0.01 (Table 6). A large influence of the type of the retraction agent on the concentration of TNF-α in saliva was proven.

Changes in TNF-α values during the study period occurred in a statistically significantly different manner only in G2, when comparing the types of the retraction agents.

## 4. Discussion

This study was based on the evaluation of the possible adverse effects of two astringents after use in the chemical–mechanical gingival retraction method, comparing them with each other throughout the entire study period.

The chemical–mechanical procedure for gingival retraction utilizes a retraction cord soaked in retraction fluid. The role of the cord is the mechanical compression of the tissue and the dislocation of the gingiva, as well as the provision of an equal concentration of the retraction agent along the entire gingival sulcus [21,22,23]. Adequate selection of the retraction cord thickness minimizes the possibility of mechanical trauma of the gingiva during the retraction procedure [21]; therefore, given the age and absence of damage to the tooth-supporting apparatus of the subjects, a small diameter cord was used (00).

Aluminium chloride is the most commonly used astringent retraction solution, with a moderate retraction effect. The concentrations of the compound are different and depend on the manufacturer. The study has proven the potential toxicity of aluminium chloride at concentrations higher than 10% [5,24,25,26]. 

Compared to aluminium-based preparations, ferric sulphate exerts the weakest retraction effect. It coagulates blood, but haemorrhage often recurs after removing the cord, and the mechanical opening of the gingival sulcus is smaller in comparison to that using aluminium salts [25]. Paudel et al. did not recommend the use of ferric sulphate at concentrations higher than 20% [26].

Phatale et al. used a pathohistological analysis to prove mild to moderate tissue damage after the use of aluminium chloride-based retraction agents [27]. Having compared the effects of various gingival retraction agents, Akca et al. concluded that tissue damage caused by ferric sulphate was significantly greater compared to that of aluminium chloride [28].

The study results indicated reversible damage of the gingival tissue after the topical application of aluminium chloride and ferric sulphate-based agents in both groups of subjects. By observing changes in the gingival tissue of the subjects after the retraction procedure and determining the GI, a mild to moderate acute inflammatory reaction of the gingival tissue one day after the application of the gingival retraction agent was revealed. The obtained results are in agreement with the findings of Phatale et al., who also found mild to moderate gingival tissue inflammation after using aluminium chloride, without tissue degeneration or necrosis [27]. Chandra et al. showed an increase in GI values after retraction procedures, favouring chemical over chemical–mechanical retraction [6]. An increase in gingival fluid volume in proportion to the increase in GI values was observed [29].

Slightly higher GI values were observed after tooth preparation, which indicated a certain mechanical tissue injury that could have accelerated the inflammatory effect of the gingival retraction agent [30]. Given the excellent regenerative capacity of the gingival tissue [31], we expected tissue healing after several days, as confirmed by GI values in the third observation period. The same conclusions were reached by Feng et al. in their pilot study, in which they demonstrated higher GI values one day after the retraction procedure, as well as a decrease in the index on the third day after the intervention, that is, complete healing after a two-week period [32].

The literature data showed adverse effects of astringent retraction agents on animals at predicted time intervals. These conclusions are consistent with the results obtained in this study and indicate the reversibility of changes in the gingival tissue [5,25,33]. The study by Akca et al. demonstrated complete healing of the gingival tissue in dogs after the application of an aluminium chloride solution [28]. Similarly, Ahmadzadeh et al. indicated complete healing of the gingival tissue in dogs seven days after the use of a ferric sulphate-based retraction agent [34].

The salivary concentration of IL-6, which is a multifunctional proinflammatory cytokine synthesized in response to tissue damage and infection, was determined. IL-6 exhibits a wide range of biological activities, including antibody production, T cell activation, B lymphocyte differentiation, and osteoclast activation [35,36].

The salivary concentration levels of IL-6 can be used for diagnostic and therapeutic purposes in regards to oral cavity diseases [37,38].

Our results are in agreement with those of other authors who have demonstrated an increase in the concentration of IL-6 in patients with periodontal damage. Increased IL-6-specific mRNA levels in mononuclear cells of the gingiva and in gingival fluid in patients with periodontitis were indicated [39]. The concentration of IL-6 in serum, saliva, and gingival fluid was higher in patients with periodontitis than in healthy subjects [40,41]. The role of IL-6 in the pathogenesis of inflammatory changes in the gingiva and the periodontium was confirmed by the researchers’ conclusion that the concentration of this cytokine in the tested samples decreased significantly after the conducted therapy [42,43]. The inhibition of the IL-6 receptor had a positive effect on the healing of patients with periodontitis and rheumatoid arthritis [44]. The same group of authors suggested that systemic and local IL-6 overproduction may play a vital role in the development of periodontal diseases [45].

TNF-α is an important mediator in inflammatory processes, autoimmune diseases, and allergic reactions [46]. This proinflammatory cytokine is the product of monocytes, macrophages, and fibroblasts [47]. The local cellular effect of TNF-α involves the adhesion of polymorphonuclear leukocytes to endothelial cells, the degranulation of polymorphonuclear cells, the activation of phagocytosis, and the expression of an intercellular adhesion molecule [47].

Our results were in agreement with the results of other authors who found high concentrations of TNF-α in the gingival fluid of patients with periodontal diseases [46,47]. Feng et al. studied the amount of TNF-α in the gingival fluid of subjects before, as well as 1, 3, 7, 14, and 28 days after, the retraction procedure. The results of the study, as in our research, showed a decrease in the studied parameter value in the interval between the first and third day, with the amount of TNF-α proportionally decreasing with time until returning to the initial level on the fourteenth day [32].

The level of TNF-α depends on the severity and degree of the disease of the tooth-supporting apparatus. Increased concentrations of TNF-α at the site of oral tissue damage prior to the appearance of the clinical signs of the disease may be found, thus making this marker particularly diagnostically important [48].

The reversibility of the resulting changes indicated a potentially low likelihood that the chemical–mechanical retraction of the gingiva would result in permanent damage to the periodontal tissue, which was supported by the findings of other authors as well. According to the literature search performed, this is the first study conducted on a carefully selected homogeneous group of subjects. The optimistic nature of the presented results could be substantiated by a longer study interval, which would probably result in the complete recovery of the gingiva, the lowest predicted values of the index for assessing the condition of the gingiva, and the absence of its recession. A combination of clinical study and laboratory testing of the potential inflammatory effect of gingival retraction agents can provide relevant conclusions that would be applicable in dental practice.

The limitations of this study include the comparison of a large number of gingival retraction agents with each other, as well as the examination of the possible pro-inflammatory effect of the chemical method of retraction compared to the standard chemical–mechanical method, by analysing various markers of inflammation. Given the higher frequency of application of the combination of gingival retraction agents and retraction cord, it was concluded that the obtained results would be more relevant for everyday clinical practice, which was proven when compared with the findings of other authors. The examined clinical parameter is widely discussed and indisputable in its diagnostic potential; as for cytokines that indicate the existence of inflammatory changes, as stated in the previous text, there are many of them, which provides the opportunity for further research into iatrogenic damage occurring during the manufacture of fixed dental restorations.

## 5. Conclusions

During the production of fixed prosthetic restorations, iatrogenic damage is possible; therefore, the possible inflammatory effect of the chemical–mechanical gingival retraction procedure during impression taking was examined using clinical parameters and the concentration of selected pro-inflammatory cytokines.

The values of the GI, as well as the salivary concentrations of TNF-α and IL-6, one day after the application of both gingival retraction agents in both study groups, were statistically significantly higher compared to the values at the beginning of the study. After 72 h, the values were lower than in the second observation period, yet still statistically significantly higher than at the start of the study, which indicated a mild to moderate reversible inflammatory reaction. Higher values of the studied parameters were observed with the application of the ferric sulphate-based retraction agent, as well as in teeth that were prepared prior to the retraction procedure. These results, analogous to the literature data, indicated the reversibility of the changes that occurred, which is optimistic data indicating that with the correct use of available dental materials and adequate clinical procedures, optimal results can be obtained. Further research will be directed towards the comparison of chemical and chemical–mechanical methods of gingival retraction by analysing other clinical parameters and markers of inflammation.

## Figures and Tables

**Table 1:** GI values, depending on the type of the gingival retraction agent used, during different observation periods.

Group	Subgroup	T0	T1	T2
G1	R1	0.33 ± 0.41 (0.00)	2.37 ± 0.52 *** (2.50)	1.27 ± 0.59 *** (1.00)
	R2	0.73 ± 0.75 (1.00)	2.30 ± 0.65 *** (2.50)	1.57 ± 0.56 *** (1.50)
G2	R1	0.17 ± 0.24 (0.00)	1.13 ± 0.52 *** (1.00)	0.43 ± 0.42 *** (0.50)
	R2	0.23 ± 0.26 (0.00)	1.83 ± 0.49 *** (2.00)	1.00 ± 0.38 *** (1.00)

The values of the continuous variables given as X ± SD (Me). ***—*p* < 0.001 (Wilcoxon signed rank test) vs. T0.

**Table 2:** Variations in IL-6 salivary concentration (pg/mL), according to type gingival retraction agent used, over the course of observation.

Group	Subgroup	T0	T1	T2
G1	R1	6.26 ± 0.85 (6.38)	12.96 ± 1.99 *** (12.79)	7.64 ± 1.09 *** (7.99)
	R2	7.34 ± 1.38 (6.90)	12.50 ± 1.84 *** (12.64)	9.54 ± 2.15 *** (8.67)
G2	R1	4.03 ± 0.61 (4.03)	5.12 ± 0.56 *** (5.04)	4.57 ± 0.63 *** (4.53)
	R2	4.12 ± 0.68 (4.06)	4.84 ± 0.77 *** (4.66)	4.54 ± 0.92 *** (4.35)

The values of the continuous variables given as X ± SD (Me). ***—*p* < 0.001 (paired-samples Student *t*-test/Wilcoxon signed rank test) vs. T0.

**Table 3:** Salivary concentration of TNF-α (pg/mL), based on the type of the gingival retraction agent used, during various observation periods.

Group	Subgroup	T0	T1	T2
G1	R1	24.09 ± 4.46 (22.05)	36.16 ± 4.24 *** (35.27)	32.04 ± 7.91 *** (30.18)
	R2	33.05 ± 5.20 (33.98)	42.11 ± 5.48 *** (42.15)	38.12 ± 5.27 *** (38.18)
G2	R1	14.59 ± 2.25 (13.78)	19.28 ± 2.57 *** (18.51)	16.12 ± 3.21 *** (15.54)
	R2	13.55 ± 2.93 (13.98)	16.32 ± 3.16 *** (16.96)	14.21 ± 2.66 *** (15.17)

The values of the continuous variables given as X ± SD (Me). ***—*p* < 0.001 (paired-samples Student *t*-test/Wilcoxon signed rank test) vs. T0.

**Table 4:** The impact of gingival retraction agents on GI values in groups G1 and G2 during the study period.

GI			Within Participant Effects			Between Participant Effects	Interaction Retraction Agent × Time
	Group		R1 + R2	R1	R2		
	G1	*p* Effect size ^¶^	<0.001 *** 0.8272	<0.001 *** 0.8554	<0.001 *** 0.7900	0.2356 0.0498	0.0938 0.0819
	G2	*p* Effect size ^¶^	<0.001 *** 0.8115	<0.001 *** 0.7122	<0.001 *** 0.8732	0.0003 *** 0.3735	0.0024 ** 0.2234

^¶^—partial eta squared (ηp2); **—*p* < 0.01; ***—*p* < 0.001.

**Table 5:** The effect of gingival retraction agents on the salivary concentration of IL-6 (pg/mL) in groups G1 and G2 during the study period.

IL-6			Effect Within Same Retraction Agent			Between Participant Effects	Interaction Retraction Agent × Time
	Group		R1 + R2	R1	R2		
	G1	*p* Effect size ^¶^	<0.001 *** 0.8888	<0.001 *** 0.8826	<0.001 *** 0.9116	0.0956 0.0960	0.0012 ** 0.2367
	G2	*p* Effect size ^¶^	<0.001 *** 0.5099	<0.001 *** 0.7564	<0.01 ** 0.3037	0.7299 0.0043	0.2815 0.0439

^¶^—partial eta squared (ηp2); **—*p* < 0.01; ***—*p* < 0.001.

**Table 6:** The influence of gingival retraction agents on the salivary concentration of TNF-α (pg/mL) in groups G1 and G2 during the study period.

TNF-*α*			Effects Within Group			Between Participant Effects	Interaction Retraction Agent × Time
	Group		R1 + R2	R1	R2		
	G1	*p* Effect size ^¶^	<0.001 *** 0.7151	<0.001 *** 0.6923	<0.001 *** 0.7772	0.0004 *** 0.3651	0.1828 0.0599
	G2	*p* Effect size ^¶^	<0.001 *** 0.6797	<0.001 *** 0.8581	0.0025 ** 0.4538	0.0467 * 0.1339	0.0386 * 0.1179

^¶^—partial eta squared (ηp2); *—*p* < 0.05; **—*p* < 0.01; ***—*p* < 0.001.

## Data Availability

The data presented in this study are available on request from the corresponding author.
